# Estimation of standardized ileal digestible calcium and phosphorus requirements of broiler chickens aged 21–35 days

**DOI:** 10.1016/j.psj.2025.106205

**Published:** 2025-12-05

**Authors:** C.W. Lee, C. Kong

**Affiliations:** a Department of Animal Science and Biotechnology, Kyungpook National University, Sangju 37224, South Korea; b Department of Animal Science, Kyungpook National University, Sangju 37224, South Korea; c Research Institute for Innovative Animal Science, Kyungpook National University, Sangju 37224, South Korea

**Keywords:** Broiler, Digestible calcium, Digestible phosphorus, Requirement, Response surface methodology

## Abstract

This study investigated the standardized ileal digestible (SID) calcium (Ca) and phosphorus (P) requirements of broiler chickens using response surface methodology (RSM). A total of 464 male Ross 308 broilers, aged 21 days, were assigned to nine treatments in a completely randomized design, with six replicates per treatment and ten replicates for the central treatment. The experimental diets incorporated SID Ca and P at five concentration levels: 3.20 and 5.90, 3.84 and 4.70, 3.84 and 7.10, 5.40 and 4.20, 5.40 and 5.90, 5.40 and 7.60, 6.96 and 4.70, 6.96 and 7.10, as well as 7.60 and 5.90 g/kg, respectively. Body weights and feed intake were recorded on days 28 and 35 to assess growth performance. Tibias were collected to analyze tibial ash, Ca and P content, and bone mineral density (BMD). Additionally, tibia bone breaking strength (BBS) was measured on day 35. RSM-based statistical analysis was performed using Design-Expert® 13 software following a central composite design. From d 21 to 28, varying SID Ca and P content did not affect growth performance (*P* > 0.05), as no quadratic response was observed (*P* > 0.05); however, tibial ash concentration and BMD exhibited quadratic responses (*P* < 0.05). From d 28 to 35, significant quadratic effects in tibial ash concentration, BMD, and BBS were identified (*P* < 0.05). Multi-objective optimization analysis demonstrated that SID Ca and P at 6.96 and 5.53 g/kg, respectively, were necessary to optimize tibial ash concentration and BMD in broilers aged 21–28 days. Furthermore, SID Ca and P levels of 6.96 and 6.47 g/kg, respectively, were required to achieve optimal tibial ash concentration, BMD, and BBS in broilers aged 28–35 days. Based on the present results, the required SID Ca:SID P ratios were 1.26 and 1.08 for 21–28-day-old and 28–35-day-old broilers, respectively.

## Introduction

Calcium (Ca) and phosphorus (P) are essential minerals for growth and skeletal integrity. Poultry diets are typically formulated based on total Ca and non-phytate P (NPP) concentrations (NRC, 1994). However, these values solely represent the quantity of minerals provided and do not reflect their actual utilization by the animals. Excessive nutrient supply potentially leads to increased excretion, resulting in wasted feed resources and environmental pollution (Omotoso et al., 2023; Rao et al., 2006). In plant-based feed ingredients, 50–85 % of P is stored as phytate, the salt form of phytic acid (Nuamah et al., 2024). In the small intestine, phytic acid readily chelates with calcium, the most abundant cationic mineral in the diet, forming an insoluble complex that poultry find challenging to hydrolyze (Moita and Kim, 2022; Nuamah et al., 2024). Furthermore, a high dietary Ca concentration can elevate intestinal pH, which may further diminish nutrient absorption (David et al., 2023b). The absorption and excretion of Ca and P in the body depend on the coordinated actions of hormones and transporters in the intestine, kidneys, and skeletal system; moreover, the bioavailability of these minerals is influenced by their dietary concentrations and the Ca:P ratio (Gautier et al., 2017; Li et al., 2017). Employing standardized ileal digestible (SID) content instead of total nutrient content in feed ingredients presents an efficient strategy to account for both the utilization capacity of animals and the interaction effects of Ca and P.

Growth performance and bone characteristics are critical factors in determining the dietary Ca and P requirements of broilers (Jiang et al., 2016). Considering that Ca and P influence each other’s bioavailability, their concentrations and the Ca:P ratio in the diet should be taken into account. While certain studies have examined the complex interactions between these minerals by treating them as independent variables (Wang et al., 2021; Wu et al., 2023), others have focused on adjusting the dietary level of only one nutrient, either Ca or P (Bai et al., 2022; Walk et al., 2022). This discrepancy possibly arises from labor and cost constraints, which increase significantly when experiments involve multiple levels of both Ca and P, along with their ratios. Response surface methodology (RSM) serves as a valuable tool for addressing these challenges. It is a statistical technique that analyzes the effects of multiple independent variables on a dependent variable simultaneously, facilitating the identification of optimal conditions. A key advantage of RSM is its ability to yield extensive information with fewer treatment combinations compared with a full factorial design. Fallah et al. (2020) successfully employed RSM to determine the Ca and NPP requirements of broilers from 1 to 21 days of age. However, few studies have assessed the Ca and P requirements of broilers during the finisher phase, and none have simultaneously estimated their SID Ca and P requirements. Therefore, the present study aimed to estimate the SID Ca and P requirements of broiler chickens from 21 to 35 days of age.

## Materials and methods

Experimental procedures were reviewed and approved by the Institutional Animal Care and Use Committee of Kyungpook National University, Republic of Korea (approval number: KNU 2024-0076).

### Animals, experimental design, and dietary treatments

Day-old male Ross 308 chicks were individually identified using neck tags and randomly housed in floor pens (1 *m* × 1 m) within an environmentally controlled room. Feed and water were provided ad libitum, and the temperature was initially set at between 32°C and 34°C during the first week and gradually reduced to 22°C by day 35. On d 21, a total of 464 birds were individually weighed and randomly allocated to nine treatments based on body weight, using a 5-level, 2-factor central composite design (CCD). The experimental diets incorporated SID Ca and P at five concentration levels: 1) 3.20 and 5.90, 2) 3.84 and 4.70, 3) 3.84 and 7.10, 4) 5.40 and 4.20, 5) 5.40 and 5.90, 6) 5.40 and 7.60, 7) 6.96 and 4.70, 8) 6.96 and 7.10, or 9) 7.60 and 5.90 g/kg, respectively. The CCD consisted of five distinct levels for each experimental factor: a center point, two intermediate (factorial) levels, and two outer axial levels. The levels of each factors were positioned at equal intervals around the central point (Diet 5: 5.4 g/kg SID Ca and 5.9 g/kg SID P). Each treatment comprised six replicates, except for the central treatment (run no. 5), which included ten replicates, with eight birds per pen. The SID Ca and P concentrations of feed ingredients were derived from previous research conducted in the same laboratory (Lee and Kong, 2024). All experimental diets were provided in mash form. A corn–soybean meal basal diet was formulated to meet or exceed the Ross 308 grower-phase nutrient specifications for essential amino acids, while crude protein and metabolizable energy concentrations were established within the range recommended between the grower and finisher phases (Aviagen, 2022). All experimental diets were isoenergetic and isonitrogenous.

### Sample collection and chemical analyses

On days 21, 28, and 35, each chicken was weighed individually, and feed leftover was recorded to calculate body weight gain (BWG) and the gain-to-feed ratio (G:F). Mortality was monitored daily to adjust feed intake values accordingly. On day 28, two birds per pen, selected based on median body weights, were euthanized via CO2 asphyxiation to collect tibias and middle toes for the analysis of bone mineral density (BMD) and ash, Ca and P concentrations. On day 35, an additional two birds with median body weights were selected from the remaining six in each pen and euthanized using the same method for further analysis of BMD, bone breaking strength (BBS) and ash, Ca and P concentrations. Tibia and toe samples were immediately stored in a freezer at −20°C until further analysis. Tibia samples were defatted by soaking in ethyl ether for 48 h, while toe samples were defatted for 6 h. All samples were subsequently dried to a constant weight in a drying oven (JSOF-150, JS Research, Gongju, Korea) at 105°C for 24 h. Left tibial BMD was determined using dual-energy X-ray absorptiometry (InAlyzer, Medikors Inc., South Korea), while right tibial BBS was measured using an Instron® Universal Testing Machine (3342, Instron Corp., Norwood, USA). All experimental diets were ground through a 1.0-mm screen using a mill grinder (CT 293 Cyclotec™; Foss Ltd., Denmark) for subsequent analysis. The samples were ashed at 550°C for 12 h to determine crude ash, total Ca and total P concentrations. Total concentrations of Ca and P were analyzed using inductively coupled plasma–optical emission spectrometry (Optima 8300, PerkinElmer, USA) in the experimental diets and tibia samples.

### Statistical analysis

Experimental data were analyzed using the MIXED procedure of SAS 9.4 software (SAS Institute Inc., Cary, NC, USA). The statistical model included dietary treatment as a fixed effect and replication as a random effect. Differences were considered statistically significant at *P* < 0.05. When dietary effects were significant, Tukey’s adjustment was used to compare mean values among treatments. The pen served as the experimental unit. Birds within a pen whose final body weights deviated by more than 1.5 times the interquartile range (IQR) were classified as outliers and subsequently excluded from the growth performance analysis, with corresponding feed intake data adjusted accordingly. For bone characteristics, values exceeding 2.5 times the IQR were considered outliers. Considering that tibia samples were pooled at the pen level, outlier detection and exclusion were conducted at the replication (pen) level rather than the individual level.

Optimization was achieved using a CCD featuring five levels of SID Ca (3.20, 3.84, 5.40, 6.96, and 7.60 g/kg) and SID P (4.20, 4.70, 5.90, 7.10, and 7.60 g/kg), analyzed with Design-Expert® 13 software (Stat-Ease Inc., Minneapolis, USA). Each pen served as the experimental unit. The experimental data were fitted to a second-order polynomial regression model represented as follows:

Y=β0+βixi+βjxj+βiixi2+βjjxj2+βijxixj+ε

where Y is the response of interest; β0 represents the intercept; βi, βj, βii, βjj, and βij symbolize the coefficients estimated by the model; xi and xj represent SID Ca and P, respectively; and ε denotes the residual associated with the experiment. The adequacy of the model was confirmed by comparing the predicted R² and adjusted R² values, ensuring that the difference between them was less than 0.2. The effect of each factor was evaluated using a desirability function and importance to determine the SID Ca and P requirements of broiler chickens. In Design-Expert, the desirability value ranges from 0 to 1, with values closer to 1 indicating more optimal outcomes. The software assigned an importance scale of 1 to 5. The importance of response variables was initially set to 5, but if a significant lack of fit was detected for a quadratic response, we reduced that response’s importance to 1 to enable multi-objective optimization calculations.

## Results and discussion

Table 1.

**Table 1:** Ingredient and chemical compositions of experimental diets on an as-fed basis.

Ingredient, g/kg	Dietary treatments								
	1	2	3	4	5	6	7	8	9
Corn	541.00	541.00	541.00	541.00	541.00	541.00	541.00	541.00	541.00
Soybean meal	339.00	339.00	339.00	339.00	339.00	339.00	339.00	339.00	339.00
Cornstarch	48.61	47.66	37.36	32.93	30.06	27.24	16.49	12.46	8.07
Soybean oil	34.06	34.50	39.27	41.32	42.65	43.96	48.93	50.80	52.83
Sodium bicarbonate	0.80	0.80	0.80	0.80	0.80	0.80	0.80	0.80	0.80
Limestone	-	6.48	-	17.02	9.64	2.24	23.23	12.80	21.45
Monocalcium phosphate	13.53	9.87	17.68	7.24	16.16	25.07	9.86	22.45	16.16
Monosodium phosphate	2.31	-	4.20	-	-	-	-	-	-
Sodium chloride	4.00	4.00	4.00	4.00	4.00	4.00	4.00	4.00	4.00
Vitamin premix^1^	1.50	1.50	1.50	1.50	1.50	1.50	1.50	1.50	1.50
Mineral premix^2^	1.50	1.50	1.50	1.50	1.50	1.50	1.50	1.50	1.50
Choline chloride	4.00	4.00	4.00	4.00	4.00	4.00	4.00	4.00	4.00
L-Arg	0.56	0.56	0.56	0.56	0.56	0.56	0.56	0.56	0.56
L-Ile	0.57	0.57	0.57	0.57	0.57	0.57	0.57	0.57	0.57
L-Lys-HCl	2.19	2.19	2.19	2.19	2.19	2.19	2.19	2.19	2.19
L-Met	2.55	2.55	2.55	2.55	2.55	2.55	2.55	2.55	2.55
L-Cys	1.62	1.62	1.62	1.62	1.62	1.62	1.62	1.62	1.62
L-Thr	1.24	1.24	1.24	1.24	1.24	1.24	1.24	1.24	1.24
L-Val	0.96	0.96	0.96	0.96	0.96	0.96	0.96	0.96	0.96
Calculated value									
AME, kcal/kg	3,070	3,070	3,070	3,070	3,070	3,070	3,070	3,070	3,070
Crude protein	205	205	205	205	205	205	205	205	205
Total Ca	3.82	5.45	4.57	8.70	7.71	6.71	11.38	9.97	11.89
Total P	6.76	5.47	8.06	4.92	6.78	8.63	5.47	8.08	6.78
Non-phytate P	4.25	2.96	5.54	2.41	4.26	6.11	2.95	5.57	4.26
SID Ca^3^	3.20	3.84	3.84	5.40	5.40	5.40	6.96	6.96	7.60
SID P^3^	5.90	4.70	7.10	4.20	5.90	7.60	4.70	7.10	5.90
SID Ca:SID P ratio	0.54	0.82	0.54	1.29	0.92	0.71	1.48	0.98	1.29
Total Ca: Non-phytate ratio	0.90	1.84	0.82	3.61	1.81	1.10	3.85	1.79	2.79
Analyzed value									
Total Ca	4.80	6.00	5.20	8.60	7.80	7.00	10.90	10.10	11.70
Total P	7.70	6.10	8.50	5.40	7.20	8.80	5.80	8.40	7.20

AME=apparent metabolizable energy; Ca=calcium; P=phosphorus; SID=standardized ileal digestible.

1 Supplies the following quantities per kilogram of diet: vitamin A, 18,000 IU; vitamin D_3_, 6,000 IU; vitamin E, 75 mg; vitamin K_3,_ 5 mg; thiamin, 5 mg; riboflavin, 13 mg; nicotinic acid, 90 mg; pantothenic acid, 30 mg; pyridoxine, 6.8 mg; cobalamin, 0.03 mg; folacin, 3.3 mg; biotin 0.33 mg.

2 Supplies the following quantities per kilogram of diet: Mn, 180 mg; Zn, 165 mg; Fe, 75 mg; Cu, 24 mg; I, 1.9 mg; Se, 0.5 mg.

3 Based on SID values determined in a previous digestibility study conducted in the same laboratory (Lee and Kong, 2024).

Only one bird died during the first experimental period (days 21 to 28), and mortality was not associated with any specific diet. Standardized ileal digestible Ca and P levels did not impact growth performance (*P* > 0.05) in broilers from 21 to 28 d of age (Table 2). Moreover, growth performance exhibited no quadratic response (*P* > 0.05), which precluded the estimation of a maximal response via RSM. In contrast, tibial ash concentration and BMD were influenced by the dietary treatments and displayed clear quadratic responses (*P* < 0.05) to SID Ca and P levels (Table 3). These findings align with those of Walk et al. (2022), who reported that in 25–42-day-old broilers, growth performance was unaffected by experimental diets with SID Ca levels ranging from 0.13 % to 0.46 %, while a quadratic effect on tibial ash percent was observed depending on the SID Ca level. The absence of an effect on growth performance may have resulted from the adaptation of the chickens to nutrient-deficient diets. Two mechanisms potentially explain this adaptation. First, the chickens may enhance nutrient utilization through improved absorption and digestibility of the deficient minerals. Second, they may mobilize minerals stored in the bones to compensate for inadequate dietary intake. Blood Ca and P concentrations remain relatively stable and are minimally influenced by dietary intake, as their homeostasis is regulated by metabolic processes in the intestines, kidneys, and bones (Li, Zhang et al., 2017). Broilers can adjust Ca and P digestibility when dietary levels are low or the Ca:P ratio is imbalanced (Angel, 2017; Yan et al., 2005). Additionally, they can increase endogenous phytase activity to enhance dietary P utilization (Davies et al., 1970). Walk et al. (2022) reported a quadratic decrease in the apparent ileal digestibility of Ca as dietary SID Ca levels increased. Collectively, these observations indicate that broilers partially adapt to mineral-deficient diets by improving nutrient digestibility and absorption. The primary regulatory factors in Ca and P metabolism include parathyroid hormone, calcitonin, and calcitriol (Proszkowiec-Weglarz and Angel, 2013). Recent studies have investigated how dietary Ca and P intake levels affect the production and mRNA expression of various transport-related factors, such as alkaline phosphatase, fibroblast growth factor 23, dentin matrix protein 1, and bone Gla protein (Cao et al., 2021; Liao, 2022). These regulatory factors help mobilize Ca and P to maintain mineral homeostasis and are closely linked to bone development. In the present study, broilers were fed a standard commercial diet until day 21, suggesting that they commenced the experiment with adequate body stores of Ca and P. Since a significant portion of absorbed Ca and P is stored in bone tissue, minerals might have been mobilized from the bones to satisfy metabolic requirements if dietary intake was insufficient during the experimental period. This assumption is reinforced by the significant differences in tibial characteristics observed among treatments, indicating that bone-related measures are more sensitive indicators of Ca and P status than growth performance.

**Table 2:** Growth performance of broiler chickens fed diets varying in standardized ileal digestible (SID) calcium (Ca) and phosphorus (P) concentrations from d 21 to 28.

	Input variables, g/kg			Output variables		
Run no. (n^1^)	SID Ca	SID P	Final BW (g)	BWG (g)	FI (g)	G:F (g/g)
1 (6)	3.20	5.90	1,493.7	535.0	781.8	0.674
2 (6)	3.84	4.70	1,492.5	534.5	720.7	0.760
3 (6)	3.84	7.10	1,504.9	538.0	775.2	0.684
4 (6)	5.40	4.20	1,460.9	495.9	744.9	0.655
5 (10)	5.40	5.90	1,476.0	494.0	748.1	0.659
6 (6)	5.40	7.60	1,498.3	523.4	767.5	0.672
7 (6)	6.96	4.70	1,470.9	518.9	765.4	0.668
8 (6)	6.96	7.10	1,489.7	549.9	785.2	0.690
9 (6)	7.60	5.90	1,477.5	524.1	767.9	0.672
RMSE			59.89	38.24	54.48	0.0670
Adjusted R^2^			−0.0523	0.0110	−0.0221	0.0125
Predicted R^2^			−0.1938	−0.1215	−0.1603	−0.1223
P values						
Diet			0.9401	0.1904	0.5501	0.2374
Quadratic source						
Sequential			0.8234	0.3580	0.5876	0.3494
Lack of fit			0.9672	0.6683	0.6836	0.1940

BW = body weight; FI = feed intake; BWG = body weight gain; G:F = gain to feed ratio; RMSE = root mean squared error.

1 Number of observations (pens) per treatment.

**Table 3:** Bone mineralization of broiler chickens fed diets varying in standardized ileal digestible (SID) calcium (Ca) and phosphorus (P) concentrations from d 21 to 28.

	Input variables, g/kg			Output variables		
Run no. (n^1^)	SID Ca	SID P	Tibial ash (%)	Ca (%)	P (%)	BMD (g/cm^2^)
1 (6)	3.20	5.90	49.51^bc^	10.78	8.56	0.150^d^
2 (6)	3.84	4.70	50.01^abc^	10.55	8.30	0.161^cd^
3 (6)	3.84	7.10	48.73^c^	10.80	8.62	0.153^d^
4 (5)	5.40	4.20	51.31^ab^	11.10	8.57	0.168^abc^
5 (10)	5.40	5.90	51.29^ab^	10.89	8.74	0.168^bc^
6 (5)	5.40	7.60	49.78^abc^	11.03	8.74	0.163^bcd^
7 (6)	6.96	4.70	51.22^ab^	10.95	8.54	0.176^ab^
8 (6)	6.96	7.10	51.56^ab^	10.82	8.59	0.170^abc^
9 (6)	7.60	5.90	51.97^a^	10.99	8.79	0.181^a^
RMSE			1.157	0.424	0.412	0.0070
Adjusted R^2^			0.2884	−0.0340	−0.0604	0.6117
Predicted R^2^			0.1888	−0.1766	−0.2054	0.5598
P values						
Diet			0.0002	0.5800	0.6626	<.0001
Quadratic source						
Sequential			0.0004	0.6712	0.8643	<.0001
Lack of fit			0.6816	0.5760	0.7078	0.4815

Ca = calcium; P = phosphorus; SID = standardized ileal digestible; BMD = bone mineral density; RMSE = root mean squared error.

1 Number of observations (pens) per treatment.

During the second experimental period (days 28 to 35), mortality was minimal, with only one bird dying. No quadratic effects were observed for growth performance (*P* > 0.05), and results are detailed in Table 4. Table 5 presents the effects of experimental diets on bone characteristics, revealing significant quadratic responses for BMD and BBS (*P* < 0.01). Tibial concentration, BMD, and BBS were influenced (*P* < 0.01) by SID Ca and P level during the 28–35 d period. Unlike the 21–28 d period, however, tibial Ca concentration showed a tendency to be affected by diet during the 28–35 d period (*P =* 0.0635). This suggests that the ability of broilers to cope with deficient diets varies with age or feeding period, presumably because of a decreased capacity of older birds to enhance digestibility during the finisher phase. The gastrointestinal tract, including the development of villi and crypts, demonstrates relatively rapid growth compared with other organs during early growth stages (Ravindran and Abdollahi, 2021). Consequently, exposure to a deficient diet immediately post-hatch is expected to adversely affect gut development. Nevertheless, in the current study, experimental diets were introduced at 21 days of age. Absorption of Ca and P occurs through both active transcellular and passive paracellular transport mechanisms (Omotoso et al., 2023). A temporary increase in the digestibility of either nutrient may arise if active transporters are upregulated in response to a deficiency. Nonetheless, the duration for which this improvement can be maintained remains uncertain. Therefore, it is plausible that the improvement in digestibility and absorption merely functioned up to a specific threshold during prolonged feeding, with the compensatory effect potentially diminishing in the later stages of the experiment. Yan et al. (2005) investigated broilers fed either a control or Ca–NPP-deficient (0.6 % Ca + 0.30 % NPP) diet during the starter (hatch to d 18) and grower (d 19 to 32) phases. Their results indicated that the group transitioning from the control diet to the Ca–P-deficient diet on day 19 did not exhibit a comparable increase in total P absorption relative to those maintained on the deficient diet. In contrast, no significant differences in BWG from day 28 or the feed conversion ratio from day 23 emerged among treatment groups. This suggests that broilers may enhance Ca and P utilization to adapt to deficiencies, particularly during early growth. However, birds transitioning from a sufficient diet to a deficient diet appear less capable of adaptation than those continuously fed a Ca- or P-deficient diet. Furthermore, Yan et al. (2005) noted that bone mineral content and BMD consistently remained the lowest across all ages in birds persistently fed the Ca–P-deficient diet, suggesting that early developmental deficiencies in Ca or P may compromise the foundation of bone formation. Conversely, Valable et al. (2018) reported that broilers on Ca- and P-deficient diets during the grower phase could recover bone mineralization with adequate supplementation of these nutrients. Notwithstanding, complete restoration to the control level necessitated appropriate adjustments in Ca and P concentrations during the repletion phase.

**Table 4:** Growth performance of broiler chickens fed diets varying in standardized ileal digestible (SID) calcium (Ca) and phosphorus (P) concentrations from d 28 to 35.

	Input variables, g/kg			Output variables		
Run no. (n^1^)	SID Ca	SID P	Final BW (g)	BWG (g)	FI (g)	G:F (g/g)
1 (6)	3.20	5.90	2,216.7	728.2	1,020.6	0.714
2 (6)	3.84	4.70	2,235.2	738.3	1,112.0	0.676
3 (6)	3.84	7.10	2,232.9	724.6	1,001.1	0.725
4 (6)	5.40	4.20	2,187.2	720.7	1,017.5	0.708
5 (10)	5.40	5.90	2,219.8	739.0	1,037.6	0.712
6 (6)	5.40	7.60	2,230.5	730.8	1,024.0	0.713
7 (6)	6.96	4.70	2,178.5	696.8	1,017.0	0.685
8 (6)	6.96	7.10	2,247.0	743.9	1,012.7	0.736
9 (6)	7.60	5.90	2,163.5	693.0	1,007.3	0.688
RMSE			92.69	44.45	78.62	0.0388
Adjusted R^2^			−0.0215	0.0479	0.0104	0.0212
Predicted R^2^			−0.1593	−0.0806	−0.1241	−0.1124
P values						
Diet			0.7818	0.4191	0.3922	0.1651
Quadratic source						
Sequential			0.7603	0.1838	0.3615	0.3012
Lack of fit			0.8404	0.8510	0.3945	0.1354

BW = body weight; FI = feed intake; BWG = body weight gain; G:F = gain to feed ratio; RMSE = root mean squared error.

1 Number of observations (pens) per treatment.

**Table 5:** Bone mineralization of broiler chickens fed diets varying in standardized ileal digestible (SID) calcium (Ca) and phosphorus (P) concentrations from d 28 to 35.

	Input variables, g/kg		Output variables				
Run no. (n^1^)	SID Ca	SID P	Tibial ash (%)	Ca (%)	P (%)	BMD (g/cm^2^)	BBS (kgf)
1 (6)	3.20	5.90	50.18^abc^	10.80	9.83	0.170^d^	33.081^d^
2 (6)	3.84	4.70	52.22^ab^	11.39	9.89	0.185^abc^	40.671^dc^
3 (6)	3.84	7.10	49.06^c^	10.89	9.52	0.175^cd^	34.933^cd^
4 (6)	5.40	4.20	49.35^bc^	10.93	9.16	0.196^a^	42.279^ab^
5 (9)	5.40	5.90	51.95^abc^	11.42	9.85	0.188^abc^	41.269^b^
6 (6)	5.40	7.60	51.17^abc^	11.15	9.42	0.192^ab^	41.299^bc^
7 (5)	6.96	4.70	52.88^a^	11.28	9.94	0.192^ab^	48.814^a^
8 (6)	6.96	7.10	52.52^ab^	11.32	10.02	0.197^a^	48.615^a^
9 (5)	7.60	5.90	53.23^a^	11.52	9.89	0.197^a^	40.589^bc^
RMSE			1.659	0.429	0.553	0.0069	3.4512
Adjusted R^2^			0.1507	0.0441	−0.0226	0.5944	0.4859
Predicted R^2^			0.0338	−0.0966	−0.1687	0.5381	0.4119
P values							
Diet			0.0004	0.0635	0.1705	<.0001	<.0001
Quadratic source							
Sequential			0.0236	0.2109	0.5791	<.0001	<.0001
Lack of fit			0.1856	0.3585	0.4188	0.1530	0.0061

SEM = standard error of the mean; BMD = bone mineral density; BBS = bone breaking strength; RMSE = root mean squared error.

1 Number of observations (pens) per treatment.

In the present study, multi-objective optimization over the 21–28-day period indicated that the optimal SID Ca and P levels for maximizing tibial ash concentration and BMD were 6.96 and 5.53 g/kg, respectively (Table 6). Nevertheless, these estimates may be slightly low, as they were derived from a restricted range of SID Ca and P dietary levels, a necessary limitation to maintain prediction accuracy. Converting these SID-based values to total dietary Ca and P requirements presents challenges owing to variability in total Ca and P concentrations resulting from the ingredient composition of the diets. Furthermore, as the requirements identified in this study were solely based on bone characteristics, direct comparisons with findings from previous studies proved difficult. The multi-objective optimization of bone mineralization—including tibial ash concentration, BMD, and BBS—demonstrated that optimal responses in broilers aged 28–35 days were achieved at 6.96 g/kg SID Ca and 6.47 g/kg SID P (Table 6).

**Table 6:** Single- and multi-objective optimization of growth performance and bone mineralization in broilers fed experimental diets from d 21 to 35.

	Input variables, g/kg		Predicted output at optimal point	Desirability
Item	SID Ca	SID P		
D 28				
Tibial ash	6.96	5.90	Maximum = 52.03 (%)	0.76
BMD	6.96	5.26	Maximum = 0.177 (g/cm^2^)	0.74
Total*	6.96	5.53	Maximum ash = 52.00 (%)	0.75
			Maximum BMD = 0.177 (g/cm^2^)	
D 35				
Tibial ash	6.96	6.37	Maximum = 51.99 (%)	0.91
BMD	6.69	6.34	Maximum = 0.200 (g/cm^2^)	0.86
BBS	6.96	7.10	Maximum = 46.319 (kgf)	0.68
Total⁎⁎	6.96	6.47	Maximum = 51.97 (%)	0.86
			Maximum BMD = 0.199 (g/cm^2^)	
			Maximum BBS = 45.169 (kgf)	

Ca = calcium; P = phosphorus; SID = standardized ileal digestible; BMD = bone mineral density; BBS = bone breaking strength.

⁎ Importance of determining SID Ca and P requirements using Design-Expert® software: tibial ash = 5; BMD = 5.

⁎⁎ Importance of determining SID Ca and P requirements using Design-Expert® software: tibial ash = 5; BMD = 5; BBS = 1.

David et al. (2023a) reported that when broilers were fed diets containing SID Ca levels ranging from 2.0 to 4.0 g/kg, both growth performance and bone characteristics increased linearly with rising dietary SID Ca levels. Owing to the linear nature of this response, the authors could not determine a precise requirement, suggesting that the highest Ca level tested remained below the required level. In the current study, while we examined higher SID Ca levels (3.2–7.6 g/kg) and observed differences in bone criteria, the absence of quadratic trend in growth responses imply that even 7.6 g/kg SID Ca might not have sufficed to reach a requirement plateau. Alternatively, the lack of growth performance responses associated with SID Ca and P concentrations may indicate that previously proposed Ca requirements have been overestimated. Furthermore, since the residual effects of diets administered prior to the experimental periods cannot be ruled out, further studies extending the experimental phase to encompass a longer duration of bird age will be essential for accurately estimating these nutritional requirements.

In summary, from d 21 to 28, growth performance was not influenced by SID Ca and P concentrations, rendering the estimation of requirements infeasible. Nonetheless, the estimated requirements for tibial ash concentration and BMD were determined to be 6.96 and 5.53 g/kg, respectively. From d 28 to 35, requirement estimates derived from multi-response optimization—considering tibial ash concentration, BMD, and BBS—were 6.96 and 6.47 g/kg, respectively. The SID Ca:SID P ratios were 1.26 and 1.08 for 21–28-day-old and 28–35-day-old broilers, respectively.

## CRediT authorship contribution statement

**C.W. Lee:** Writing – review & editing, Writing – original draft, Validation, Software, Investigation, Formal analysis. **C. Kong:** Writing – review & editing, Writing – original draft, Visualization, Validation, Supervision, Software, Resources, Project administration, Methodology, Investigation, Funding acquisition, Formal analysis, Data curation, Conceptualization.

## Disclosures

The authors declare the following financial interests/personal relationships which may be considered as potential competing interests:

Changsu Kong reports financial support was provided by Rural Development Administration. If there are other authors, they declare that they have no known competing financial interests or personal relationships that could have appeared to influence the work reported in this paper.
